# Evaluation of a proposed mixture model to specify the distributions of nuchal translucency measurements in antenatal screening for Down's syndrome

**DOI:** 10.1258/jms.2010.009108

**Published:** 2010-03

**Authors:** J P Bestwick, W J Huttly, N J Wald

**Affiliations:** Wolfson Institute of Preventive Medicine, Barts and the London Queen Marys School of Medicine and Dentistry, Charterhouse Square, London EC1M 6BQ, UK; Wolfson Institute of Preventive Medicine, Barts and the London Queen Marys School of Medicine and Dentistry, Charterhouse Square, London EC1M 6BQ, UK; Wolfson Institute of Preventive Medicine, Barts and the London Queen Marys School of Medicine and Dentistry, Charterhouse Square, London EC1M 6BQ, UK

## Abstract

**Objectives:**

A mixture model of crown–rump length (CRL)-dependent and CRL-independent nuchal translucency (NT) measurements has been proposed for antenatal screening for Down's syndrome. We here compare the efficacy of the mixture model method with the standard method, which uses NT multiple of the median (MoM) values in a single distribution.

**Settings:**

A routine antenatal screening programme for Down's syndrome comprising 104 affected and 22,284 unaffected pregnancies.

**Methods:**

The ability of NT to distinguish between affected and unaffected pregnancies was compared using the mixture model method and the standard MoM method by using published distribution parameters for the mixture model of NT and parameters derived from these for the standard MoM method. The accuracy of the two methods was compared for NT and maternal age by comparing the median estimated risk with the prevalence of Down's syndrome in different categories of estimated risk.

**Results:**

Using NT alone observed estimates of discrimination using the two methods are similar; at a 70% detection rate the false-positive rates were 12% using the mixture model method and 10% using the MoM method. Risk estimation was marginally (but not statistically significantly) more accurate using the standard MoM method.

**Conclusions:**

The mixture model method offers no advantage over the standard MoM method in antenatal screening for Down's syndrome, is more complicated and less generalizable to other data-sets. The standard MoM method remains the method of choice.

## INTRODUCTION

It has been proposed that nuchal translucency (NT) measurements (in mm) in antenatal screening for Down's syndrome be fitted to two distributions in affected pregnancies and two distributions in unaffected pregnancies at each crown–rump length (CRL) measurement – a mixture model.^1^ It was suggested that this model better describes NT measurements than the standard method of using a single distribution of NT for affected pregnancies and a single distribution for unaffected pregnancies at each day of gestation (with NT expressed as multiple of the median [MoM] values and gestational age estimated from CRL) but this has not been shown. The mixture model method assumes that in the majority of unaffected pregnancies NT increases with increasing CRL, while in the remainder NT is constant, and conversely in the majority of affected pregnancies NT is constant with increasing CRL, while in the remainder NT increases. The mixture model method involves estimating a relatively large number of NT distribution parameters (means, standard deviations and proportions that are CRL-dependent and CRL-independent) compared with the standard MoM method. This is described in Appendix A.

A concern with the use of a mixture model to describe the distribution of NT measurements is that the model may be too tailored to the data-set from which it was derived and may not be generalizable to other data-sets. This concern and the lack of comparison with the standard MoM method in the report proposing its use^1^ prompted us to perform a quantitative comparison of the two methods using an independent data-set to determine whether the mixture model offers an improvement in antenatal screening for Down's syndrome.

## METHODS

We compared the proposed mixture model method with the standard MoM method using data on the 104 Down's syndrome and the 22,284 unaffected pregnancies screened at the Wolfson Institute of Preventive Medicine with CRL measurements between 45 and 84 mm (between 11 + 0 and 13 + 6 weeks gestation discussed in our accompanying paper in this issue of the *J Med Screen*;^2^ two affected and 356 unaffected pregnancies with CRL measurements less than 45 mm were excluded because no parameters were specified in the proposed mixture model for CRL measurements less than 45 mm.^1^

In the proposed mixture model among both Down's syndrome and unaffected pregnancies, NT is dependent on CRL in some pregnancies and independent of CRL in others. A mixture of a CRL-dependent Gaussian distribution and a CRL-independent Gaussian distribution is used to describe NT measurements in both affected and unaffected pregnancies at each CRL measurement.

### Unaffected pregnancies

1. In the majority of unaffected pregnancies NT depends on CRL, with a log-quadratic relationship used to describe the change in NT with increasing CRL. The standard deviation of NT is assumed independent of CRL. This generates a Gaussian distribution at each CRL, with a different mean but the same standard deviation;
2. In the remainder of unaffected pregnancies NT is independent of CRL, so the same Gaussian distribution is used across the range of CRL.

### Down's syndrome pregnancies

1. In the majority of affected pregnancies NT is independent of CRL, so the same Gaussian distribution is used across the range of CRL;
2. In the remainder of affected pregnancies NT is dependent on CRL and the distributions at each CRL are assumed to be the same as the CRL-dependent distributions in unaffected pregnancies.

Ninety-four percent of Down's syndrome pregnancies follow the CRL-independent distribution. In unaffected pregnancies the proportion decreases with increasing CRL, from about 12% at a CRL of 45 mm to 3% at a CRL of 84 mm. The 10 parameters of a mixture model (five for affected – 2 means, 2 standard deviations and the proportion for one or other distribution, and 5 for unaffected) are estimated simultaneously, which can be done in various ways. One method selects different combinations of the parameters for affected pregnancies and ‘converges’ on the combination that fits the data most closely. The same is repeated for unaffected pregnancies.

The parameters (means and standard deviations) for the single distributions of NT MoM values in affected and unaffected pregnancies at 11, 12 and 13 completed weeks were derived from the mixture model parameters for CRL measurements that correspond to these gestational ages (49, 62 and 76 mm, respectively^3^) using integration methods (see Appendix B).

Screening performance of the two methods were compared, by applying the two sets of parameters (mixture model and MoM) to data on the 104 Down's syndrome and the 22,284 unaffected pregnancies. For each pregnancy, the likelihood ratio for each method was calculated and detection rates for specified false-positive rates and false-positive rates for specified detection rates calculated. The accuracy of risk estimation of the two methods using NT and maternal age was compared using a validation method previously described.^4^ Categories of risk were defined by quintiles of risk in affected pregnancies (so that there are approximately equal numbers of affected pregnancies in each category) and the prevalence of Down's syndrome in each category is tabulated with the median estimated risk in each category.

## RESULTS

**Figure 1:**
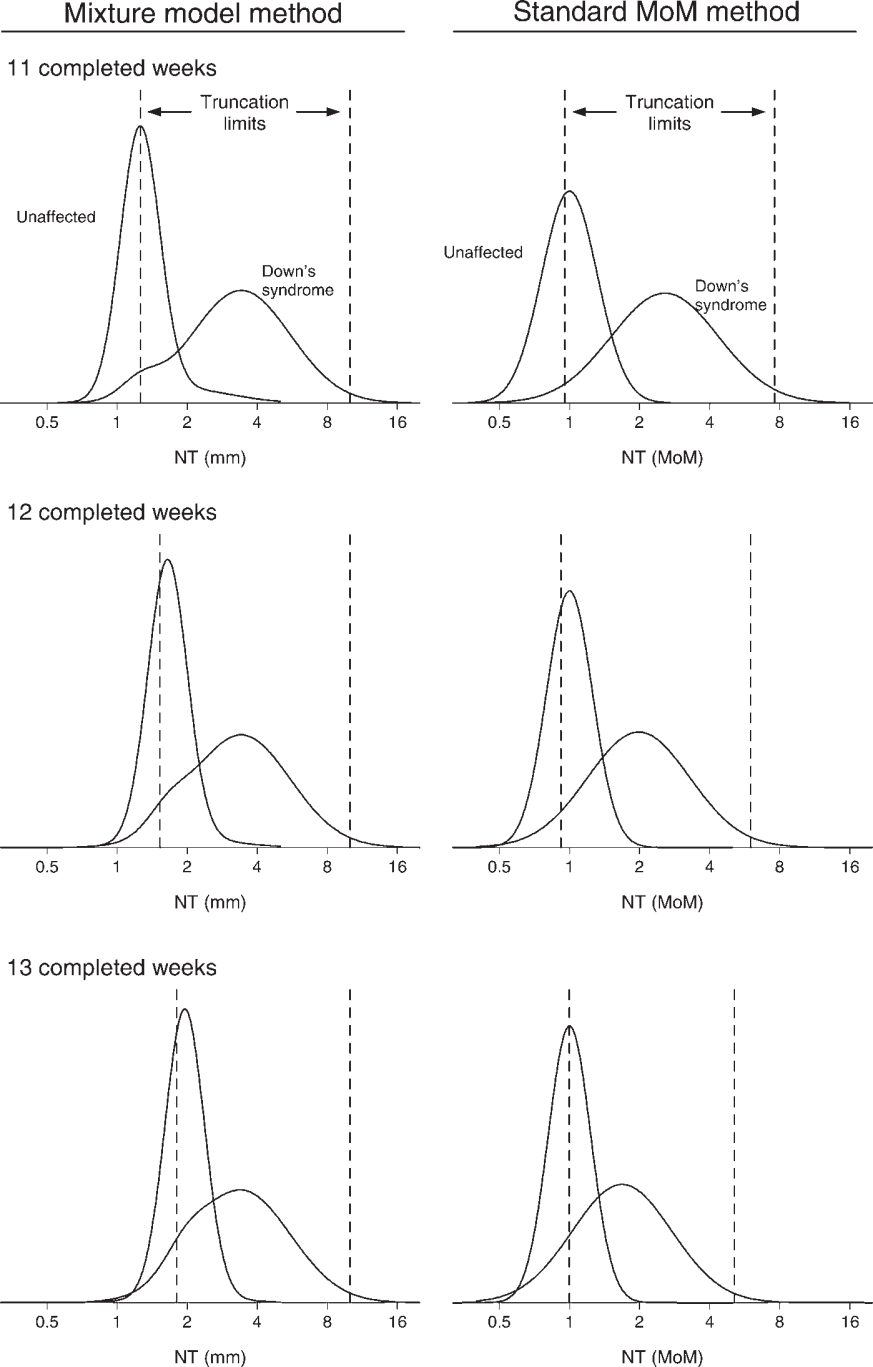
Mixture model distributions of nuchal translucency (NT) in mm and distributions of NT multiple of the median (MoM) values in Down's syndrome and unaffected pregnancies at 11, 12 and 13 completed weeks' gestation. Truncation limits shown (vertical lines) are those specified by Wright *et al.*^1^

Figure 1 shows the relative frequency distributions of NT in Down's syndrome and unaffected pregnancies at 11, 12 and 13 completed weeks' gestation together with truncation limits using the mixture model method and the standard MoM method (the parameters [means and standard deviations] of the mixture model method distributions and standard MoM method distributions are given in Appendix B, Table B1). With the mixture model method the distribution in affected pregnancies remains approximately stationary from week to week while the distribution in unaffected pregnancies moves to the right as gestation increases. With the standard MoM method the distribution in affected pregnancies moves to the left with increasing gestation while the distribution in unaffected pregnancies remains stationary. Figure 1 shows that the mixture distributions are similar to the MoM distributions, i.e. since the proportion of unaffected pregnancies that have CRL-independent NT and the proportion of affected pregnancies that have CRL-dependent NT are low the mixture distributions closely resemble the Gaussian distributions of NT MoM values.

Table 1 shows the screening performance of using NT alone with the mixture model method and with the standard MoM method, using an independent data-set, i.e. a data-set not used to derive the distribution parameters. There is a marginal improvement in screening performance using the standard MoM method.

**Table 1:** Screening performance of NT alone (measured between 11 and 13 weeks gestation), based on 104 Down's syndrome and 22,284 unaffected pregnancies according to method

	DR (%) for FPR of				FPR (%) for DR of				
Method	1%	3%	5%	10%	50%	60%	70%	80%	90%
Mixture model method^1^	41	57	62	68	1.9	4.3	12	28	43
Standard MoM method*	42	56	63	68	1.7	4.1	10	20	41

NT, nuchal translucency; DR, detection rate; FPR, false-positive rate; MoM, multiple of the median

*Derived from the mixture model distributions^1^

Table 2 compares the accuracy of risk estimation of the two methods using NT and maternal age. The MoM method provides the more accurate risk estimates. The estimated risk using the mixture model approach is lower than the prevalence in each category but this bias is not evident using the standard MoM method. However, the differences are small and could be due to chance. The MoM method pulls risk estimation further apart than the mixture model method; the median risk was one in 26 in Down's syndrome pregnancies and one in 2114 in unaffected pregnancies using the MoM method, compared with one in 53 and one in 1858, respectively, using the mixture model approach, supporting the conclusions seen in Table 2.

**Table 2:** NT and maternal age: observed prevalence of Down's syndrome and median estimated risk in categories define by quintiles of risk of Down's syndrome in affected pregnancies

Mixture model method				Standard MoM method			
Risk category	Number of Down's syndrome pregnancies	Median expected risk of Down's syndrome	Prevalence of Down's syndrome	Risk category	Number of Down's syndrome pregnancies	Median expected risk of Down's syndrome	Prevalence of Down's syndrome
≥1 in 2.9	21	1 in 1.9	1 in 1.6	≥1 in 1.0	21	1 in 1.0	1 in 1.7
1 in 2.9	21	1 in 12	1 in 6.4	1 in 1.0	21	1 in 3.1	1 in 6.8
1 in 26	21	1 in 70	1 in 20	1 in 9.2	21	1 in 32	1 in 17
1 in 111	21	1 in 223	1 in 97	1 in 58	21	1 in 184	1 in 90
≤1 in 322	20	1 in 2249	1 in 988	≤1 in 308	20	1 in 2429	1 in 997

NT, nuchal translucency; MoM, multiple of the median

Single distribution NT MoM parameters derived from the mixture model NT (mm) parameters are unlikely to be exactly the same as those derived directly from the raw data because methods of estimation are usually used that avoid the influence of outliers. For this reason we performed a sensitivity analysis, comparing screening performance by increasing or decreasing the derived median MoM in Down's syndrome pregnancies by 20%, and by increasing or decreasing the standard deviations in Down's syndrome and unaffected pregnancies by 20%. The results of the sensitivity analysis were consistent with our finding that screening performance is not improved with the mixture model method.

## DISCUSSION

The mixture model distributions in affected and unaffected pregnancies and the standard MoM distributions are similar (see Figure 1) so there is no reason to expect them to yield materially different screening performances. In both approaches, as gestational age (or CRL) increases the distributions in Down's syndrome and unaffected pregnancies become closer; with the mixture model method the unaffected distribution moves closer to the almost stationary affected distribution while with the standard MoM method the affected distribution moves closer to the stationary unaffected distribution. When both methods were applied to an independent data-set, screening performance was marginally better using the standard MoM method with a single distribution, for example to achieve a 70% detection rate for NT alone the false-positive rate is 12% using the mixture model and 10% using the standard MoM method. The accuracy of risk estimation was marginally better using the MoM approach, with estimated risks calculated from the mixture model being lower than the observed prevalence within each risk category. The sensitivity analysis indicated that our results are robust to imprecision in estimating the distribution parameters of NT.

If it is known that the distributions of NT are different in pregnancies with or without a factor other than Down's syndrome, then it is valid to construct a mixture model with separate means and standard deviations for pregnancies with and without such a factor among pregnancies with and without Down's syndrome. In the absence of data on such an external factor the assumption of two distributions based only on the distribution itself can be misleading – tailoring a distribution too closely to the study sample. It is unlikely to be generalizable to other data-sets. If a distribution appears to have a ‘hump’ in one of its tails, further research should be performed to explain the reason for this and adopting a complex model avoided unless it is shown to be necessary.

This study shows that the more complex mixture model has no advantage over the standard MoM method in antenatal screening for Down's syndrome.

## Figures and Tables

**Figure A1:**
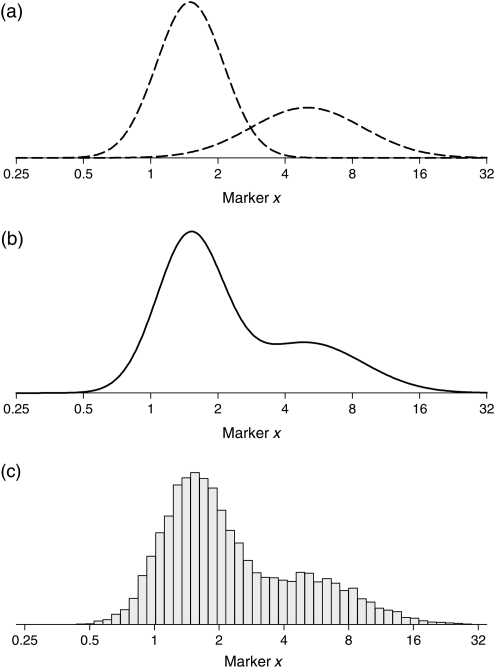
Two Gaussian distribution curves of hypothetical screening marker *x* (a), mixture distribution curve (b) and histogram of the marker that might be observed in affected individuals (c)

## APPENDIX A

### Description of the mixture model

The mixture model method assumes that a variable, *x*, comes from two (or more) sub-populations in affected individuals and two (or more) in unaffected individuals. It is not possible to categorize the sub-populations other than from the distribution of *x*. To illustrate the method we consider a hypothetical marker *x*, and for simplicity illustrate this in affected individuals only. Two distributions are fitted to a data-set of *x* in affected individuals, with a proportion (*p*_1_) of observations belonging to one distribution and a proportion (*p*_2_) belonging to the other. The resulting combined (mixed) distribution is defined by five parameters (2 means, 2 standard deviations and *p*_1_ [not *p*_2_ since *p*_2_ = 1 − *p*_1_]). In practice with the inclusion of unaffected individuals there would be 10 parameters.

Figure A1 shows two fitted Gaussian distribution curves of a hypothetical marker *x* in affected individuals (a), the mixture distribution (b) and what might be observed to justify the use of a mixture distribution, presented as a histogram (c). The five parameters are estimated simultaneously and this could be performed using maximum-likelihood estimation; the mixture distribution defined by the five parameters, which provides the best fit to the data maximizes the product of the likelihood (height of the curve) for each value of *x*. Methods such as the expectation maximization algorithm can be used to estimate the parameters or other methods, such as the Markov Chain Monte Carlo procedure can be used.

After defining a mixture distribution for a measurement in affected and unaffected individuals, the risk of being affected is calculated in the same way as with the standard single distribution method; that is by multiplying the risk of being affected before having the measurement (expressed as an odds) by the likelihood ratio obtained from the mixture model distributions in affected and unaffected individuals. As with the standard single distribution method, the likelihood ratio for a value *m* of the screening marker is the height of the affected mixture distribution curve (Figure A1(b) in the hypothetical example above) at *m* divided by the height of the unaffected mixture distribution curve at *m*. Calculating a weighted average of the likelihood ratio from the CRL-dependent Gaussian distribution and the likelihood ratio from the CRL-independent Gaussian distribution using the proportion of pregnancies attributed to each distribution as the weights would give an identical result.

## APPENDIX B

### Calculation of median NT MoM in Down's syndrome pregnancies

Given the probability density function (pdf, equation of the curve) of a Gaussian distribution is

where μ is the mean and *σ* the standard deviation, then the pdf of a mixture distribution is

with *p*_1_ the proportion of observations belonging to one distribution and 1 − *p*_1_ the proportion belonging to the other distribution. The theoretical median of a mixture distribution can be found by integrating the mixture distribution between minus infinity and *m* (or between *m* and infinity). The median can then be found by letting the integral equal to one-half and solving for *m*, i.e. the median is calculated by finding the value of *x* for which the area under the mixture distribution curve to the left and the right of this value is equal to one-half. Formally this is written as

This requires solving numerically since the solution to the integral is not defined. By using the above equations to find the median NT in both Down's syndrome (*m*_D_) and unaffected pregnancies (*m*_U_), the median MoM in Down's syndrome pregnancies is calculated as *m*_D_/*m*_U_ (by definition the median MoM in unaffected pregnancies is equal to one).

### Calculation of the standard deviation of (log_10_) NT MoM in Down's syndrome and unaffected pregnancies

The overall standard deviation of the mixture of two Gaussian distributions can be found by integrating the second moment of the mixture distribution

where *μ* = *p*_1_*μ*_1_ + (1 − *p*_1_)*μ*_2_ is the overall mean (not to be confused with the overall median) and *σ* the overall standard deviation. This does have a solution, so does not require approximation methods:

In words this can be expressed as the square root of the weighted average of the two squared means plus the weighted average of the two squared standard deviations minus the overall mean squared. Standard deviations calculated from the 2.5th to 97.5th centile interval would produce estimates similar to those using the equation above. The means and standard deviations of the mixture model method distributions and standard MoM method distributions are shown in Table B1.

**Table B1:** Parameters for overlapping distributions in Down's syndrome and unaffected pregnancies according to method

	Mixture model method (NT, mm)				Standard MoM method (NT MoM)	
	Down's syndrome		Unaffected		Down's syndrome	Unaffected
Parameter	CRL dependent	CRL independent	CRL dependent	CRL independent		
**Median**						
11 completed weeks	1.29		1.29		2.50	
12 completed weeks	1.70		1.70		1.92	
13 completed weeks	1.96		1.96		1.67	
**Standard deviation (log_10_)**						
11 completed weeks					0.2289	0.1165
12 completed weeks					0.2180	0.0969
13 completed weeks					0.2136	0.0911
**Proportion**						
11 completed weeks			0.8993	0.1007	–	–
12 completed weeks			0.9359	0.0641	–	–
13 completed weeks			0.9613	0.0387	–	–

NT, nuchal translucency; CRL, crown–rump length; MoM, multiple of the median
